# Examination of Theory of Mind, Cognitive Functions and Acute Period Clinical Features in Patients Diagnosed With Substance Use-Related Psychotic Disorder

**DOI:** 10.1192/j.eurpsy.2025.784

**Published:** 2025-08-26

**Authors:** C. Yildirim, N. Zorlu, B. Bagci, A. Bilge Bektas

**Affiliations:** 1 Psychiatry, Izmir Katip Celebi University Ataturk Education and Research Hospital; 2 Psychiatry, Izmir City Hospital, Izmir, Türkiye

## Abstract

**Introduction:**

There are recent studies examining the cognitive functions, depressive symptoms, social cognitive characteristics (Terms of Theory of Mind) and acute symptoms of patients with substance-induced psychosis, as well as varying effects of different substances on development of psychosis (Beckmann et al. Child Adolesc Psychiatr CNA.2020;131-143).

**Objectives:**

It was aimed to examine sociodemographic characteristics, clinical symptoms in acute psychotic period, cognitive functions, social cognition characteristics of hospitalized patients diagnosed with substance related psychosis; compare the severity of clinical symptoms, improvement in acute period symptoms with treatment, cognitive functions and social cognitions according to substance (methamphetamine, cannabis, polydrug).

**Methods:**

34 hospitalized patients in acute psychotic episode diagnosed with Substance-Induced Psychosis according to DSM-5 were included in study. Socidemographic Data Form, Reading the Mind in the Eyes Test, Stroop Test, Calgary Schizophrenia Depression Scale and every 3 days during the treatment PANSS (Positive Negative Syndrome Scale) were administered.

Statistical analyses done with R program. Kruskal-Wallis test was used to compare numerical variables between groups. For the significant differences between groups, Bonferroni correction was applied in post hoc analyses. P-value of less than 0.05 was considered statistically significant for all analyses. Chi-square test was used to analyze categorical variables.

**Results:**

It was determined that drug-induced psychosis patients were mostly male, unemployed, young adults, started using drugs before the age of 18, lived in irregular urban areas. Methamphetamine, cannabis and polydrug-related psychosis were compared; There was no difference in cognitive functions and social cognition characteristics between all groups. At the end of treatment, it was observed that the rate of improvement in positive and negative symptoms and the decrease in PANSS scores were greatest in methamphetamine group, there was no difference between cannabis and polydrug groups. We found that PANSS and negative symptom severity before starting treatment were not different between methamphetamine and cannabis, and initial positive symptoms were more severe in methamphetamine than cannabis.

**Conclusions:**

Methamphetamine group showed greater improvements in positive and negative symptoms and total PANSS scores compared to cannabis and polydrug. The fact that there was less improvement in cannabis group than in methamphetamine group supports the data in the literature identifying the relationship between cannabis and chronic psychosis (D’Souza et al, The World Journal of BP, 2022, 719–742).

No difference was found between social cognitions and cognitive functions of 3 groups.

**Keywords:**

Drug Induced Psychosis, Methamphetamine, Cannabis, Theory of Mind.

**Disclosure of Interest:**

None Declared

